# How removing visual information affects grasping movements

**DOI:** 10.1007/s00221-018-5186-6

**Published:** 2018-02-05

**Authors:** Chiara Bozzacchi, Eli Brenner, Jeroen B. Smeets, Robert Volcic, Fulvio Domini

**Affiliations:** 1 Center for Neuroscience and Cognitive Systems@UniTN, Istituto Italiano di Tecnologia, Rovereto, Italy; 2 Department of Human Movement Sciences, Vrije Universiteit, Amsterdam, The Netherlands; 3 Department of Psychology, New York University Abu Dhabi, Abu Dhabi, UAE; 4 Department of Cognitive, Linguistic and Psychological Sciences, Brown University, Providence, RI USA; 5 Social Brain Lab, The Netherlands Institute for Neuroscience, Royal Netherlands Academy of Arts and Sciences (KNAW), Meidergdreef 47, 1105 AZ Amsterdam, The Netherlands

**Keywords:** Grasping, Visual feedback, Action planning, Visual occlusion, Movement trajectory

## Abstract

Our interaction with objects is facilitated by the availability of visual feedback. Here, we investigate how and when visual feedback affects the way we grasp an object. Based on the main views on grasping (reach-and-grasp and double-pointing views), we designed four experiments to test: (1) whether the availability of visual feedback influences the digits independently, and (2) whether the absence of visual feedback affects the initial part of the movement. Our results show that occluding (part of) the hand’s movement path influences the movement trajectory from the beginning. Thus, people consider the available feedback when planning their movements. The influence of the visual feedback depends on which digit is occluded, but its effect is not restricted to the occluded digit. Our findings indicate that the control mechanisms are more complex than those suggested by current views on grasping.

## Introduction

Grasping an object is an effortless action that belongs to our everyday routine. Its apparent ease is at odds with the complexity of the underlying transformations. Numerous visual cues are used to estimate the object’s shape and egocentric position, to establish where on its surface to place one’s digits. This information is used to plan the action, which is then monitored online through visual feedback about the hand and finally through haptic feedback when touching the object (Jeannerod 1984; Smeets and Brenner 1999; Bingham et al. 2007; Domini and Caudek 2013; Volcic et al. 2013; Bozzacchi et al. 2014; Bozzacchi and Domini 2015, 2016; Volcic and Domini 2016). In the present study, we aim to specifically investigate the role of visual feedback in more detail, to establish how and when such feedback affects the kinematics of the movement.

Literature on grasping suggests that visual feedback plays an important role in the late part of the action (Gentilucci et al. 1994; Churchill et al. 2000; Fukui and Inui 2013; Rand et al. 2007). In fact, the digits’ velocity profiles are asymmetrical. Digits slow down more gradually as they approach the object than they speed up when the movement starts. This probably increases the extent to which visual feedback is available to guide the movement, because the use of feedback is limited by the motor delays that are involved (at least 100 ms; Brenner et al. 1997; Smeets et al. 2006). In the absence of visual feedback of the moving hand, people take other precautions to help their digits reach their destination in a safe manner, for instance increasing grip aperture and moving more slowly. The latter primarily involves slowing down even more near the end of the movement (Carlton 1981; Jeannerod 1984; Prablanc and Pelisson 1990; Jakobson and Goodale 1991; Gentilucci et al. 1994; Connolly and Goodale 1999; Winges et al. 2003; Watt and Bradshaw 2003; Whitwell et al. 2008).

Based on the classical reach-and-grasp view on grasping, these adjustments take place in what is defined as the closure phase of the movement, in which the hand is maneuvered around the object (Jeannerod 1984, 1988). The reach-and-grasp view postulates that the hand is transported towards the object (transport component) and once in proximity, the finger and the thumb open more and then close around the target (grip component). This view on grasping considers the hand as a grasping apparatus and, as such, experiments have always manipulated the visibility of the whole hand to study the effect of feedback on the different movement components (Jeannerod 1988; Hoff and Arbib 1993; Jakobson and Goodale 1991; Whitwell et al. 2008; Fukui and Inui 2013).

Smeets and Brenner (1999) formulated an alternative view on grasping based on independence between the digits during the movement. The double-pointing view makes no distinction between reaching and grasping components, suggesting that these are epiphenomena of both digits being transported simultaneously but independently (Smeets and Brenner 1999, 2001; Smeets et al. 2010; Verheij et al. 2012; Volcic and Domini 2014; Schot et al. 2017). It also postulates that the movements of the digits in grasping are controlled online, thus ascribing strong relevance to visual feedback (Smeets et al. 2002, 2016; Voudouris et al. 2013).

However, as the visual feedback of each digit is important only for the movement of that specific digit, visual occlusion or manipulation of one digit should leave the path of the other one unperturbed. In a recent experiment aiming to investigate this hypothesis, Melmoth and Grant (2012) pointed to an alternative additional view where digits present a separate but also hierarchical function in grasping. In their study, they tried to isolate the effect of the visibility of the single digits, using a glove to provide selective information about the thumb or the index finger in separate blocks of trials. They found that occlusion of the thumb caused longer movement times, whereas occlusion of the index finger gave rise to wider grip apertures. These findings fostered the view that the digits might be independent and play different roles in grasping movements, possibly with the thumb guiding the movement, rather than seeing the hand as a ‘single tool’ (Wing et al. 1986; Haggard and Wing 1997; Melmoth and Grant 2012, but see; Cavina-Pratesi and Hesse 2013).

The above-mentioned studies focused on the kinematic aspects occurring later in the movement, and how they changed as a result of hindered visual feedback. Their emphasis was on maximum grip aperture, peak velocity and movement duration, implicitly suggesting that no important alteration was present in the early phase of the movement. To our knowledge, no study has investigated whether what is visible while people plan their movement influences the movement. Whitwell and colleagues investigated to what extent knowing whether or not vision would be occluded during the movement affects grasping movements. What their participants saw while planning the movements was always the same, but on some trials the participants’ vision was occluded as soon as the hand started moving. Grip aperture was smaller when feedback had been available on previous trials than when it had not (Whitwell and Goodale 2009), but knowing that visual information would be available on the next trial did not make a difference (Whitwell et al. 2008). In the present study, we aim to investigate the role of partial occlusions of the trajectory to the target that are known from before the hand starts to move on the movements of the individual digits. We want to study how the movements develop across the whole trajectory when the whole movement is planned for the prevailing circumstances.

To examine how planned movements of the individual digits are tuned to the precise circumstances on each trial, we made it evident that certain digits would be occluded, either together or individually, before the movement started. We analyzed the whole movement trajectory, describing how each digit responded to the visual occlusion, rather than focusing on a single parameter such as maximal grip aperture. We expected that people would increase the margin of safety when faced with uncertainty by increasing the grip aperture. Whether this happens as the result of an equal deviation of both digits from their original path, or only due to the deviation of the occluded digit, is the main question of this study.

## Materials and method

This study consists of four experiments that were very similar to each other in their procedure. Each experiment consisted of a single session with four conditions: no occlusion (none), index finger occluded, thumb occluded and both digits occluded. Since the design of each experiment depended on the results of the preceding experiment(s), we here describe the aspects of the methods that were common to all experiments, and we describe the aspects that differed between the experiments when we introduce each experiment before presenting its results.

### Participants

In total, 40 participants volunteered to take part in the study. Some of them participated in more than one experiment and four participants took part in all four experiments. There were 20 participants in total per experiment. All signed an informed consent form prior to taking part in the experiment. Participants were right-handed, based on self-report of hand preference, and had normal or corrected to normal vision. The study was part of a research program that was approved by the ethical committee of the Faculty of Human Movement Science of the VU University, Amsterdam.

### Materials and calibration

Participants were seated with their head stabilized by a chin rest. In front of them was a table with small indentations to make it easy to place objects at specific positions. Above the table was a large (70 × 70 cm) horizontal surface that rested on four, 24.5 cm high columns that were far enough apart for the participants to be able to move their arm freely below the surface. A monitor (59.5 × 33.3 cm) that had been dismantled to remove the background and lighting, so that one could see through it when it was set to ‘white’, was embedded in this surface. A computer controlled the transparency of regions of this screen, and thereby the different feedback conditions. Bright lights below the surface, illuminating the area between the table and the semi-transparent surface, compensated for the fact that even when the screen was set to white it only let through a small part of the light. The use of partly transparent images on a screen allowed us to let our participants view the actual hand grasping a real target object while occluding specific parts of the information, rather than having to rely on some form of virtual reality.

The starting position of the hand was with the index finger and thumb contacting each other at the top of a 6-cm high cylinder. This cylinder was 15 cm to the right of the center of the participant’s body, 40 cm lower than the participant’s eyes and 30 cm from the participant’s chest. The task was to move from this position, along the fronto-parallel plane, to grasp a sphere (25–60 mm diameter) that was positioned some distance (30–50 cm) to the left of the starting position. The size of the sphere and the distance to the sphere differed between the four experiments. We chose relatively large distances so that the movements would take long enough to make use of visual feedback when possible. The movements of the thumb and index finger of the participants’ right hands were recorded at 250 Hz with an Optotrak 3020 motion tracking system (Northern Digital, Waterloo, ON, Canada).

**Fig. 1:**
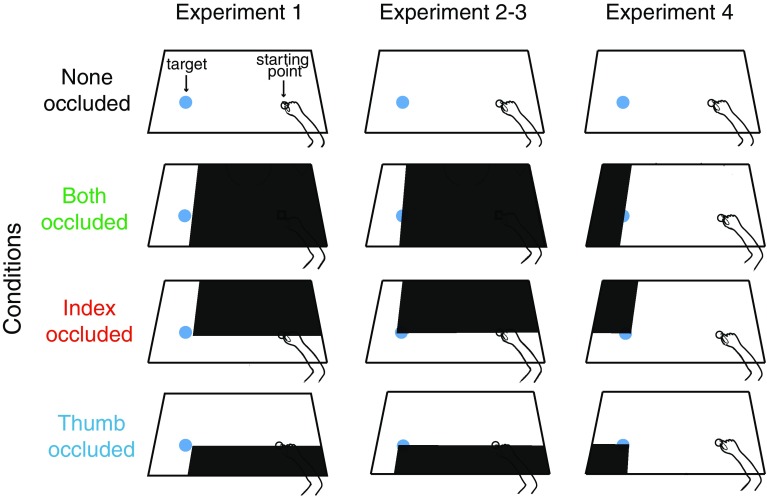
Experimental setup and experimental conditions. A computer-controlled screen was used to block vision of selected parts of the workspace (black areas). Other parts of the screen were transparent, giving participants vision of the scene, including their own hand, the target object (left blue sphere) and the starting position of the hand (right black square). There were four different conditions for each of the four experiments. When the occlusion included the contact points on the object (experiments 2–4) a small part of the object was left visible to allow participants to reach the right location

A session started with a calibration procedure. First, we determined the position of the thumb and index finger pads with respect to three infrared-emitting diodes attached to small triangular surfaces that were fixed to the nail of each finger. This was done by measuring the positions of those diodes in combination with an unattached diode held in the finger pad (see Bozzacchi and Domini 2015 for details). Next, we determined the position of the starting position by moving the unattached diode to it. After this, a horizontal line appeared on the screen and participants were asked to adjust the height of the chair so that the line passed through the center of the starting point and the center of the target object. This adjustment was used to align the borders of the occluding surfaces on the semi-transparent screen to block vision of the same part of the table for all participants (Fig. 1).

### Procedure

In each experiment, each of the 20 participants performed a single block of trials. This block included trials for the four visibility conditions. There were 10 trials for each combination of condition, object size and object distance that was to be used in the analysis. We used additional target distances and sphere sizes to prevent participants from repeating the same movement pattern on all trials. During the course of experimentation, we changed the way in which we introduced such variation, but all the experiments included the distance of 40 cm to ensure that we could compare the effects of variation of visual feedback across experiments. The trials were presented in random order.

Participants were asked to move their hand from the starting position to the sphere, grasping it with their thumb and index finger placed along the horizontal axis orthogonal to the movement direction, and to lift the sphere. At the start of each trial, the screen was completely opaque. When the experimenter started the trial, the screen became completely or partly transparent (depending on the condition). This was the signal for the participant to move the hand towards the sphere and lift it. After 3 s, the screen became opaque again indicating the end of the trial. One second later the screen turned transparent again, so that participants could find their way back to the starting position. When the digits were within 2 cm of the starting position, the screen turned opaque and a new trial could start.

### Data analysis

We investigate how the hand deviated from the path taken with full visual feedback (none condition), meaning that the screen turned fully transparent. We first considered the transport and grip components of the movement and then focused on the deviation of the single digits’ trajectories. In the latter case, we focused on the differences between the trajectories of each digit in conditions with and without occlusion. The dependent measures analyzed were: (1) differences between the transport trajectory in the none condition and the other three conditions; (2) differences between the changes in grip aperture throughout the trajectory in the none condition and the other three conditions; (3) differences between the single digits’ trajectories in the none condition and the other three conditions; (4) the maximum grip aperture; and (5) the movement duration.

**Fig. 2:**
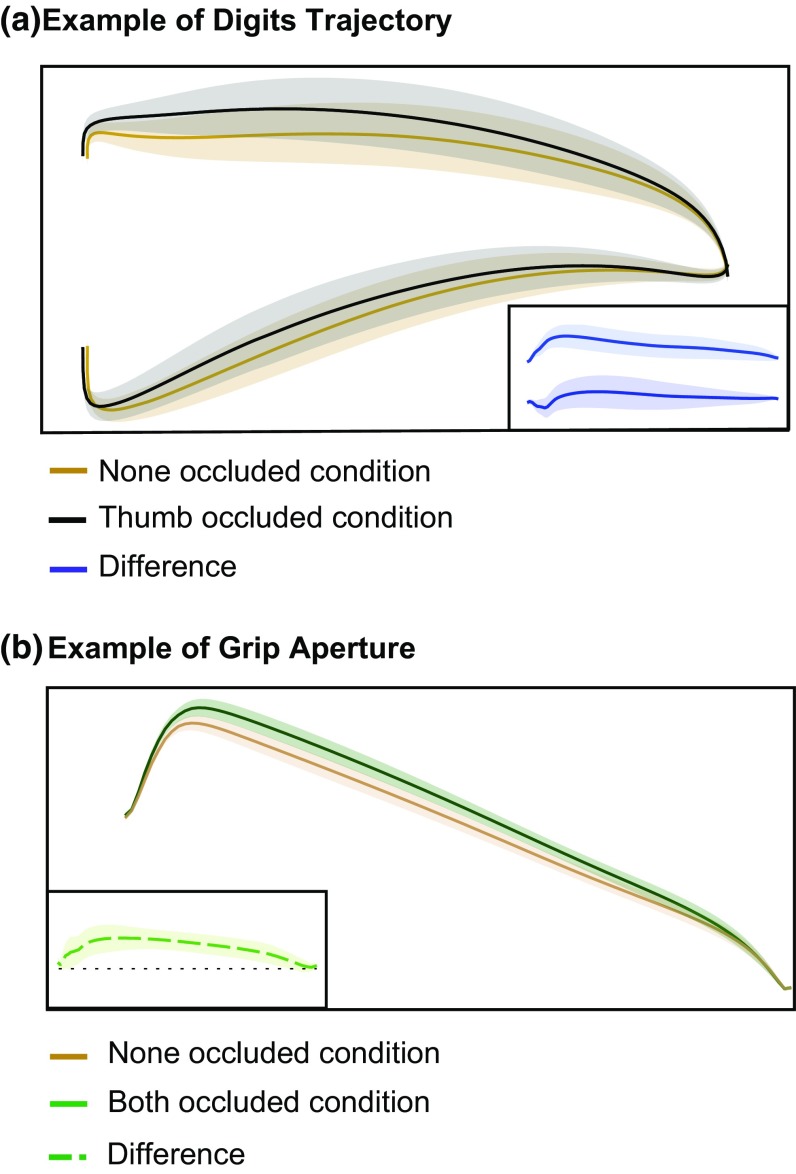
Example trajectories. **a** The averaged trajectories of the two digits of an example participant for two conditions: the thumb occluded condition (black curves) and the none occluded condition (yellow curves). The inset shows the difference between the two conditions for the same data (blue curves). **b** The averaged grip aperture for the same example participant in the both occluded condition (green curve) and the none occluded condition (yellow curve). The inset shows the difference between the conditions (green dotted curve). Shaded areas indicate 95% confidence intervals (across participants’ mean values)

To analyze how the action unfolded throughout the movement, the trajectories of the transport and each digit were spatially normalized using the total length of the movement. The trajectories were resampled to 100 points, evenly spaced along the three-dimensional trajectory from movement onset to movement end, using cubic spline interpolation. As a measure for the transport of the hand, we used the 3D trajectory of the midpoint between the tips of the digits, rather than that of the wrist (Smeets and Brenner 1999). The trajectory of the thumb, that has also been considered as a measure of transport of the hand (Wing et al. 1983; Haggard and Wing 1997; Melmoth and Grant 2012) is also provided because we provide the trajectories of the individual digits. The time-course of the grip aperture was obtained by calculating the Euclidian distance between the digits at each of the 100 resampled points of the transport trajectory (Fig. 2). This method was previously used in Volcic and Domini (2016).

For each experiment, we averaged the different variables of interest across participants. We plotted this mean difference as a function of the normalized displacement, with a shaded area representing the 95% confidence interval across participants. This means that if the shaded area does not overlap with zero, removing visual feedback affects the trajectory consistently across participants with *p* < 0.05 at that position. Positive values of the difference indicate an adjustment away from the body with respect to the none condition, whereas negative values indicate an adjustment towards the body.

To relate our results to previous studies, we also analyzed the maximum grip aperture (MGA) and the movement duration. MGA was defined as the maximum Euclidian distance between the fingertips in individual trials. Finally, movement duration was calculated as the time from movement onset to the moment participants started lifting the object (movement offset). Movement onset was determined as the moment of the last minimum thumb velocity value just prior to the first continuously increasing value of thumb velocity. Movement offset was determined on the basis of the Multiple Sources of Information method (Schot et al. 2010) combining velocity of the thumb and index finger, velocity and acceleration of the grip aperture and position of the target object in the vertical plane. For both variables, we ran a repeated measure ANOVA with condition as the main factor.

## Experiment 1

In the first experiment, participants were asked to grasp a sphere made of opaque glass (diameter 40 mm) located at two possible distances from the starting point (40 and 50 cm). Although the formal analysis was carried out on the 40 cm distance only, we confirmed that behavior at the 50-cm distance was not evidently different. Black surfaces were displayed on the screen at positions at which they occlude the digits’ movements from the starting position, while not occluding the object and its contact points (Fig. 1). Therefore, the digits were invisible until they were closer than about 30 mm to the center of the object (10 mm from the object surface). The 20 participants (11 female) performed a single block of 80 trials: 10 for each combination of condition and distance. The total duration of the experiment was about 20 min.

### Results

#### Transport and grip

**Fig. 3:**
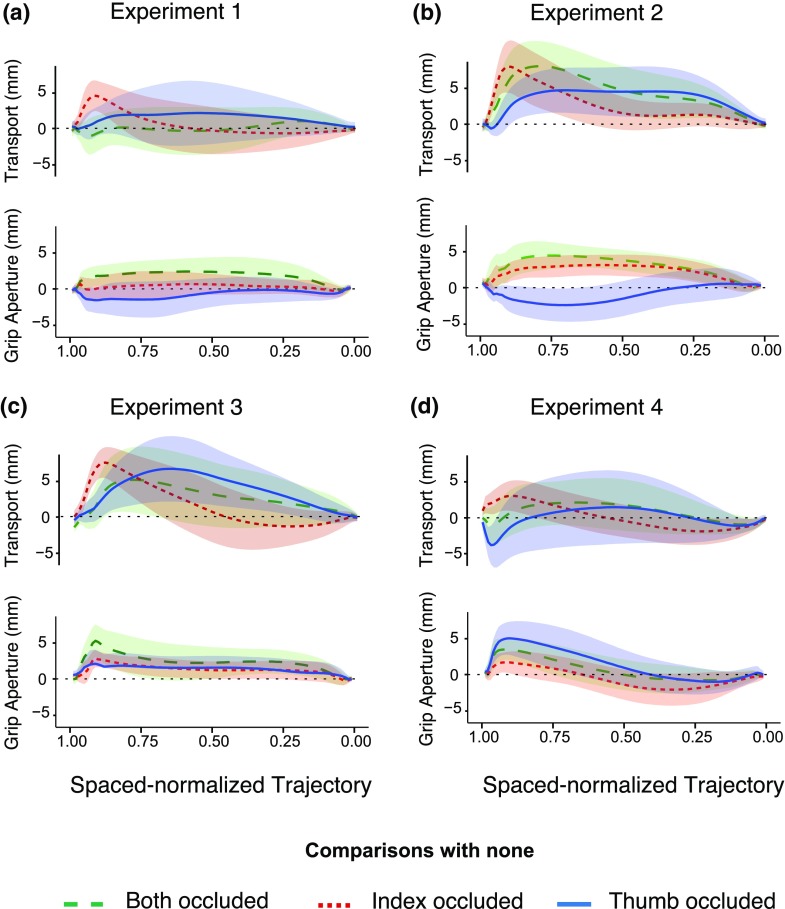
The effects of occlusion on transport and grip. For each panel, the upper plots show the difference in the transport component along the trajectory between the none occluded condition and the other experimental conditions. The lower plots show the difference in grip aperture along the trajectory between the none occluded condition and the other experimental conditions. Shaded areas represent the 95% confidence intervals (across participants’ mean values)

Occluding most of the hand’s movement path, leaving the object fully visible, affects the trajectory of the movement very modestly (Fig. 3a). The hand only deviates from the original path when the index finger is occluded (red curve). It does so in the late part of the movement. When both digits were occluded (green curve), the grip aperture tended to increase. However, the same effect was not visible when a single digit was occluded; the grip aperture even tended to decrease in response to occlusion of the thumb (blue curve).

#### Digits’ deviations

**Fig. 4:**
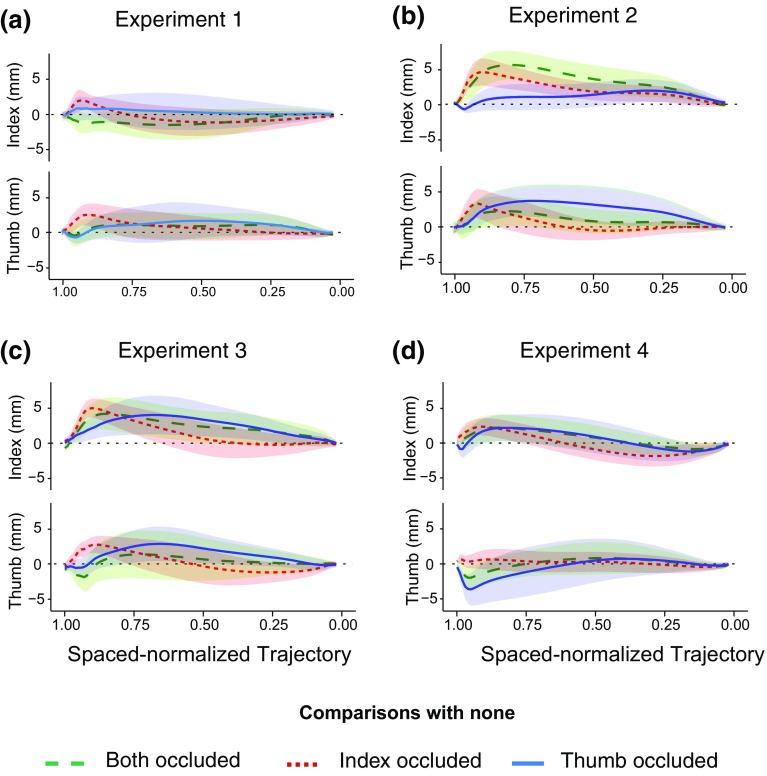
The effects of occlusion on digits’ trajectories. For each panel, the upper plots show the difference in the index finger trajectory between the none occluded condition and the other experimental conditions. The lower plots show the difference in the thumb trajectory between the none occluded condition and the other experimental conditions. Shaded areas represent the 95% confidence intervals (across participants’ mean values)

Occluding one or both digits did not have a systematic effect on the trajectory in the early part of the movement (Fig. 4a). A possible difference between the influence of occluding the index finger and thumb is in the opposite direction than we had anticipated: when removing visual feedback about the thumb, the thumb tended to deviate away from the body. When occluding the index finger, the index finger tended to deviate towards the body. These effects correspond with decreasing rather than increasing the safety margin. Near the end of the movement, both digits tended to move away from the body when the index finger was occluded.

#### Maximum grip aperture and movement duration

**Fig. 5:**
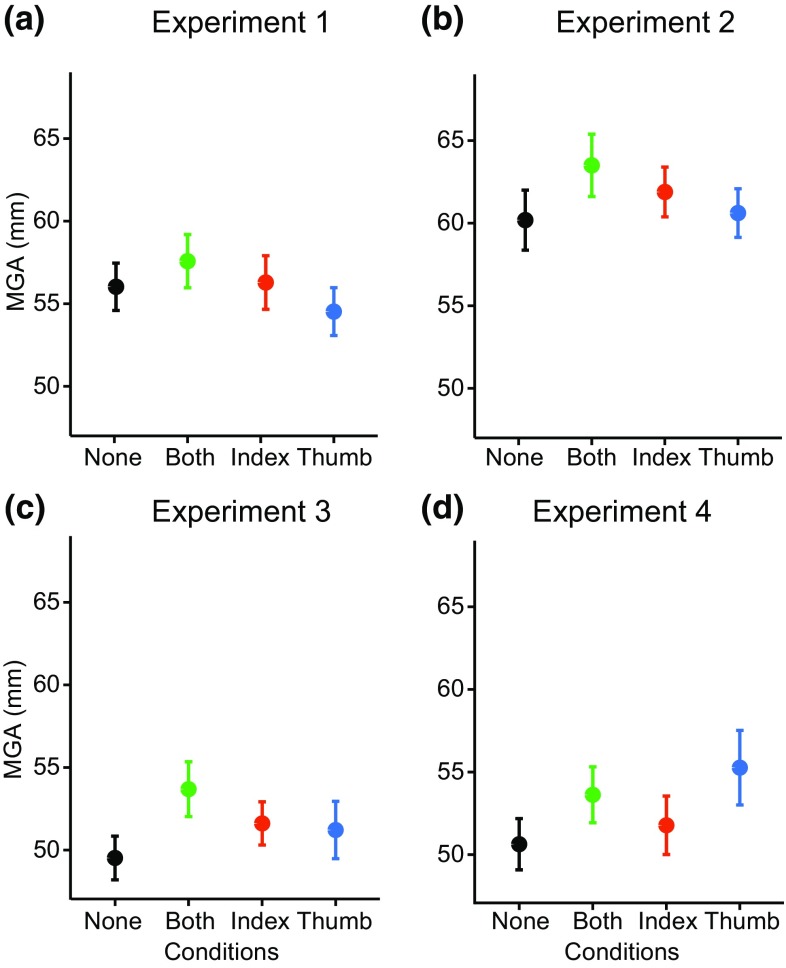
Maximum grip aperture (MGA). Maximum grip aperture for the different conditions in the four experiments. Error bars represent the standard error of the mean (across participants’ mean values)

**Fig. 6:**
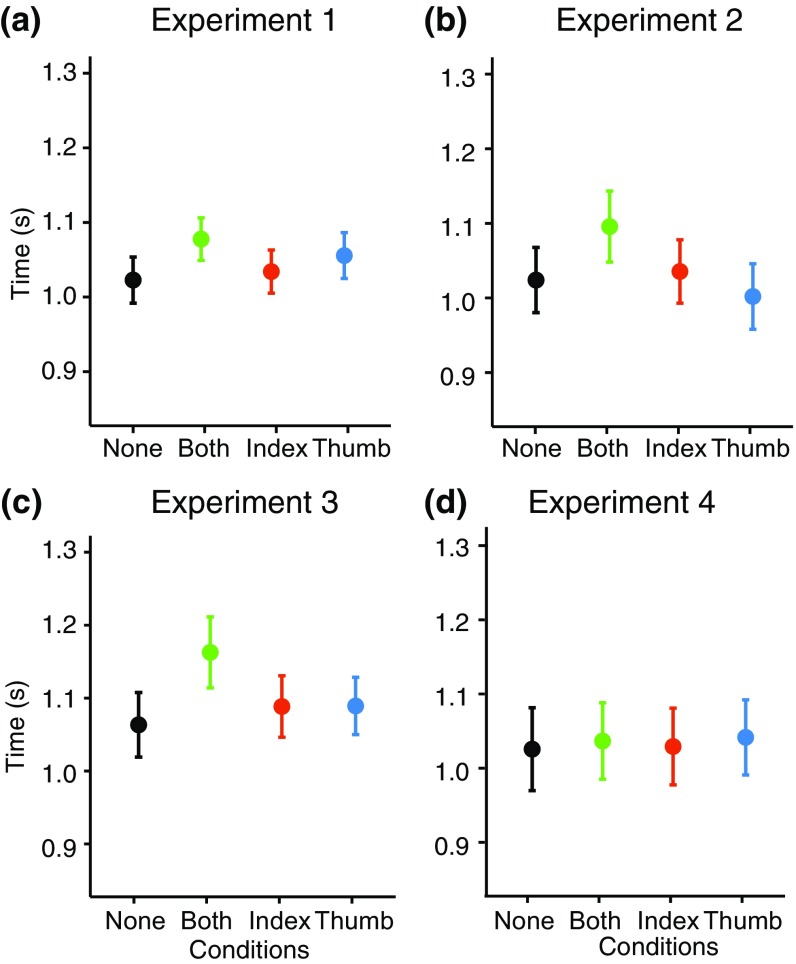
Movement duration. Total movement duration for the different conditions in the four experiments. Error bars represent the standard error of the mean (across participants’ mean values)

We separately analyzed the MGA and the movement duration by running two repeated-measured ANOVAs with condition as the main factor. For the MGA, we found a significant effect of condition [*F*(3,57) = 5.81; *p* = 0.0015]. As shown in Fig. 5a, maximum grip aperture was particularly large when both digits were occluded, and particularly small when only the thumb was occluded. Similarly, for the movement duration we found a significant main effect [*F*(3,57) = 11.71; *p* < 0.0001]. The movement was slowest when both digits were occluded (Fig. 6a).

### Discussion

We observed only small effects of the occlusions on the movement trajectories and the grip aperture did not show any significant modulation. Overall, selectively occluding either the thumb or index finger appeared to have some influence on the trajectories of both digits, not only the movement of the occluded digit, but any influence was very small.

Although we did not find the spatial effects that we predicted for either of the two views, our participants did slow down when information was removed. To our knowledge, none of the hypotheses predict to what extent people will move more slowly and to what extent they will increase their grip aperture or have their digits follow more curved paths. It is conceivable that the amount of occlusion influences their choice.

In the first experiment, the full visibility of the object included the visibility of the digits once they were enclosing the object, which might be a critical feature in guiding the movement. This might make it unnecessary to adjust the movement path. To account for this possibility, we conducted a second experiment in which the occlusion areas were extended so that the contact point on the object was also covered (Fig. 1).

## Experiment 2

The same 40-mm diameter glass sphere was used, located at two possible distances from the starting point (30 or 40 cm). Although the formal analysis was carried out on the 40-cm distance only, we confirmed that behavior at the 30-cm distance was not evidently different. In this experiment, the occlusion was extended leftward to also cover the digits’ contact points on the sphere. The left edge of the occluding area was chosen such that when both digits were occluded, both contact points were occluded, but a small part of the leftmost edge of the sphere remained visible so that participants were always aware of the position of the sphere. As in experiment 1, 20 participants (12 females) performed a single block of 80 trials combining the four conditions and the two distances.

### Results

#### Transport and grip

The extension of the occlusion to cover the contact points on the target resulted in stronger effects on both transport and grip. Figure 3b shows that in all conditions, the hand tended to shift away from the body from the very beginning of the movement. This effect was particularly strong when the thumb (blue curve) or both digits (green curve) were occluded. Occluding the index finger (red curve) led to an adjustment later in the trajectory. The modulation of the transport phase was accompanied by a modulation of the grip aperture. With respect to the none condition, occlusion of the index finger or of both digits gave rise to the expected larger grip aperture (Fig. 3b). Surprisingly, the opposite behavior was observed when the thumb was occluded.

#### Digits’ deviations

Occluding the digits as well as their contact points influenced the digits’ trajectories similarly, but more clearly than only occluding the digits (Fig. 4b). Both digits moved away from the body. The effect was stronger for the digit that was occluded than for the non-occluded one. Performance when both digits were occluded was quite similar to that when only the index finger was occluded.

#### Maximum grip aperture and movement duration

We ran a repeated measure ANOVA on the MGA and found a significant main effect of condition [*F*(3,57) = 13.84; *p* < 0.0001]. The influence of occlusion on MGA was similar to that of experiment 1, but the MGAs themselves were overall larger (Fig. 5b). Movement duration analysis showed a significant effect of condition [*F*(3,57) = 10.72; *p* < 0.0001]. Occluding both the digits (and their contact points) slowed down the movement (Fig. 6b).

### Discussion

The results of this experiment show a similar but stronger effect of the occlusion. The digits did not deviate in opposite directions, as both models would predict. Instead, both digits deviated in the same direction (causing a change in transport), with one digit deviating further in that direction, giving rise to the change in grip aperture. Very surprisingly, occluding the thumb leads to a reduction of the grip aperture. This cannot be explained in relation to the reach-and-grasp view. In the double-pointing view, this increase can only be explained if one assumes that there is also a tendency to deviate away from the occluding surface. In that case, the reduced grip aperture could be a side-effect of a larger deviation away from the occlusion for the thumb (which is occluded) than for the index finger (which is not occluded).

Although the ways in which the manipulations influenced the trajectories seem to be robust, the magnitude of the effects is modest. Could this be because we only used a single object throughout the experiments? When participants cannot see the object properly, and repeatedly grasp the same object, they might rely on prior experience instead of on vision to guide their movement. Haptic feedback is undoubtedly involved in such guidance, because haptic feedback is known to play a role in calibrating movements (Bingham et al. 2007; Schenk 2012). We, therefore, conducted a third experiment using multiple objects sizes.

## Experiment 3

In this experiment we used the same visual manipulations as in experiment 2, but with an increased number of targets and distances. We used four different wooden spheres (25, 37, 50 and 60 mm diameter) that could each be presented at one of three possible distances (30, 40 or 50 cm from the starting position). There were 10 trials for each condition in which the sphere with the diameter of 37 mm was located at 40 cm (comparable to the combination of sphere size and distance that we analyzed in the previous experiments), and a strongly reduced number of trials per condition for all other combinations: two per size (except 37 mm) for the distances of 30 and 50 cm, and one per size (except 37 mm) for the distance of 40 cm. The 20 participants (13 females) ran a total of 100 trials, which took about 30 min. Different objects were presented at different distances in a randomized order.

### Results

#### Transport and grip

The effects on the transport trajectories in experiment 3 were similar to those in experiment 2. Any occlusion caused a deviation away from the body (Fig. 3c). This effect is present from movement onset when the thumb is occluded (alone or together with the index finger). If only the index finger is occluded, only a later modulation of the transport was evident. The effects on the grip aperture trajectories differed considerably from those in experiment 2. In this experiment, all three experimental conditions led to an increased grip aperture with respect to the none condition, as anticipated. The increase started early in the movement. The effect was strongest when both digits were occluded, but was also evident in the other two conditions.

#### Digits’ deviations along the trajectory

When a single digit was occluded, both digits initially veered away from the side of the occlusion, especially when the thumb was occluded (Fig. 4c). When the index finger was occluded, both digits later veered away from the body. The magnitudes of the effects were similar to those of experiment 2, so apparently the extent to which the size and position of the sphere is varied, and properties such as the spheres’ weight (the wooden sphere was much lighter), did not have much impact on this result. The deviations in the individual digits’ trajectories (and therefore also in the transport) were more clearly consistent across the first three experiments than was the grip aperture.

#### Maximum grip aperture and movement duration

A repeated-measures ANOVA showed a significant effect of condition [*F*(3,57) = 8.58; *p* < 0.0001]. The difference between conditions resembled the one found in the previous experiments, but with overall smaller values for the maximum grip aperture in experiment 3 (Fig. 5c). The reduction in Maximal grip aperture is larger than the reduction in the sphere’s diameter (3 mm: 37 rather than 40 mm). Movement duration was also significantly different for the different conditions [*F*(3,57) = 9.99; *p* = 0.0001], with a similar pattern as was observed in the previous 2 experiments (Fig. 6c).

### Discussion

In terms of effects on the movements of the individual digits, as well as the transport, the results of this study mainly confirmed the findings of experiment 2. Increasing the variation in the size and distance of the target might have made some effects of occluding the digits slightly clearer (Fig. 4c), but the magnitudes of the effects remained quite modest. The pattern of the effects appears to be very consistent, even though the effects are small and not what we would expect from any of the views mentioned in the introduction. None of the views predicted that both digits would initially veer away from the occluding surface, as seems to be the case.

For the grip aperture, the results look qualitatively different for the different experiments. In the third experiment, we no longer found the reduction in grip aperture throughout a large part of the trajectory when the thumb was occluded, as we had found in the first and second experiment (Fig. 3a, b), or the reduction in maximal grip aperture when the thumb was occluded, as we had found in the first experiment (Fig. 5a). Based on all theories, one would expect a larger maximum grip opening when more is occluded. The fact that the maximum grip aperture was considerably smaller in experiment 3 than in experiment 2 (Fig. 5) is partly due to the use of a 3-mm smaller sphere, although the difference (6–11 mm) is larger than one would expect from sphere size alone. The remaining difference might be related to the movement times being slightly longer (Fig. 6) but it might also partly be due to the sphere’s weight being lower and relatively small within the set of spheres that were presented.

An important feature of the results of all three experiments is that there appear to be different influences of occlusion at different stages of the movement. It appears that occluding the digits influences the initial part of the movement towards the sphere and the final approach of the sphere in different ways. If the final approach primarily depends on seeing the digits near the sphere, we might be able to isolate the late component of the effect of occlusion by only manipulating visibility near the contact points, leaving the hand visible during the rest of the movement. In this case, we expect to find no effect on the early part of the trajectory, but the digits might curve differently as they reach the object.

## Experiment 4

In this experiment, we only occluded vision of the contact points, leaving the rest of the trajectory completely visible. We used the same set of objects and distances as in experiment 3. The 20 participants (eight females) performed a single 30-min block of 100 trials, with 10 trials per condition for the 37-mm wooden sphere located 40 cm from the starting point. Due to technical problems during the recording phase, one participant’s data had to be removed from the analysis.

### Results

#### Transport and grip

As in experiment 1, no consistent modulation of the transport of the hand was found in the early phase of the movement, but in the second half of the movement the hand showed an overall tendency to move away from the occlusion (Fig. 3d). At the very end of the movement, when the hand was about to reach the object, the transport was significantly modulated by the single-sided occlusions. The hand moved towards the occluded side. Grip aperture was only enlarged in the second half of the trajectory when only vision of the contact points was removed (in all conditions).

#### Digits deviation along the trajectory

Occluding only the contact points had a slightly more specific effect on the digit of which the contact point was occluded than occluding the digits during the whole movement (Fig. 4d). Occluding the contact point of the thumb mainly influenced the thumb’s trajectory. The thumb’s trajectory deviated from its trajectory in the none occluded condition by moving closer to the body. Occluding the index finger’s contact point caused the index finger to move further away from the body, without influencing the thumb’s trajectory. When both digits’ contact points were occluded, each digit responded by moving in opposite directions, with the thumb moving closer to the body and the index finger further away.

#### Maximum grip aperture and movement duration

A repeated measures ANOVA on the MGA showed a significant effect of condition [*F*(3,54) = 11.25; *p* < 0.0001]. The grip aperture mainly increased in the conditions in which the thumb was occluded (Fig. 5d). Movement duration showed no significant effect of condition [*F*(3,54) = 0.59; *p* = 0.63] (Fig. 6d).

### Discussion

The results of the final experiment are consistent with the idea that there are two separate effects of occluding the digits’ trajectories. The early phase of the movement is sensitive to the visibility of the digits, whereas the late part of the movement is primarily affected by visibility of the contact points.

## General discussion

In this study, we investigated the effect of visual feedback of the hand and object on grasping movements, focusing on the initial part of the movement and thus on the importance of visual feedback on action planning. To this aim, we separately manipulated the visibility of the two digits and of their contact points, to investigate whether the hand adjusts by symmetrically opening the digits, or whether only the perturbed digits change their behavior.

Our findings suggest that there are two different effects of visual feedback on grasping movements. The (partial) occlusion of the object has an effect on the later part of the movement. On the other hand, an extended occlusion of visibility of the whole hand’s or of single digits’ paths affects the movement from the very beginning. Our results seem to suggest a way of controlling grasping more different and complex than those previously described in the literature.

Previous studies suggest that visual feedback is likely to have most impact on movements when the hand is about to interact with the object (Carlton 1981; Jeannerod 1984; Prablanc and Pelisson 1990; Volcic and Domini 2016). This is consistent with studies showing gaze shifting to the object that is to be grasped and remaining there as the movement unfolds (de Grave et al. 2008; Cavina-Pratesi and Hesse 2013; Voudouris et al. 2016), and the effect of blocking vision of the contact points that we report. Here, we show in addition that occluding the trajectory also affects the early part of the movement. This influence on the initial part of the trajectory is different from that in the late part. It is characterized by the digits moving away from the occluding surface (blue curves generally curving upwards and red curves curving downwards in Fig. 4). The tendency to veer away from the side of the occluding surface was too small to substantially change the extent to which the digits were visible (for a similar small effect see Voudouris et al. 2012) and might be the result of making sure to avoid possible obstacles at places that one cannot directly see (Voudouris et al. 2016) rather than of trying to see the digits themselves.

The grip aperture was not always increased by the occlusion, except when both digits were occluded from the start. In this case, grip aperture was larger from the beginning of the movement. The influence of occluding the individual digits differed across the experiments in a manner that cannot simply be accounted for on the basis of whether the digits, the contact points, or both were occluded.

Analyzing the digits separately highlighted the fact that the modulation of the trajectory by blocking online visual feedback differed for the two digits. Participants do not simply move their digits in a safer way (open their grip more) the more the trajectory is occluded. Moreover, their adjustments are not limited to the digit that is occluded. The results of experiments 1, 2 and 3 clearly show that both digits are affected by occluding either digit. The different behavior adopted for each kind of occlusion might reflect various strategies to optimize the limited information available, rather than only adopting a wider margin of safety.

The conclusion that there are multiple components to the choice of the path is consistent with the differences between the experiments. When visual feedback of the trajectory was unobstructed (experiment 4), occluding either the thumb’s or the index finger’s contact points had a more specific effect on the final part of the occluded digit’s movement. In particular, when the contact point of the index finger was occluded, the thumb did not deviate from its original path, in accordance with the view that considers the two digits independently (Smeets and Brenner 1999; Verheij et al. 2012).

In conclusion, the present findings highlight the importance of distinguishing between visual feedback of the hand and object, as they influence the movement at different stages and in different ways.
